# Amorphous/Nanocrystalline Carbonized Hydrochars with Isomeric Heterogeneous Interfacial Polarizations for High-performance Microwave Absorption

**DOI:** 10.1038/s41598-019-48926-3

**Published:** 2019-08-27

**Authors:** Yujie Qi, Dongchao Wei, Gui-Mei Shi, Mu Zhang, Yang Qi

**Affiliations:** 1 Institute of Materials Physics and Chemistry, School of Materials Science and Engineering, Northeastern University, Shenyang, Liaoning 110819 P.R. China; 2 College of Science, Shenyang University of Technology, Shenyang, Liaoning 110870 P.R. China; 3 Key Laboratory for Anisotropy and Texture of Materials, School of Materials Science and Engineering, Northeastern University, Shenyang, Liaoning 110819 P.R. China

**Keywords:** Materials for energy and catalysis, Nanoscale materials

## Abstract

Carbon allotropes and their derivatives have exhibited superior performances for microwave absorption ascribed to their dielectric attenuation capacity raised from the multiple dipolar configurations. Such characteristics could be achieved by constructing diverse micro/nanoscale architectures, incorporating vacancies and heteroatoms, and composing with heterogeneous components. Alternatively, we herein present a facile approach for the synthesis of carbonized hydrochars, which are composed of dispersed ultrafine nanocrystallines graphite and amorphous matrix. Such a isomeric construction has a high-density interfaces, accompanied with significant interfacial polarizations, leading to the improvement of microwave absorbing capabilities. For carbonized hydrochars, the RL_min_ value can reach −40.36 dB, and corresponding effective bandwidth is 2 GHz. This work provides a concept for designing microwave absorption materials based on isomeric heterogeneous interfacial polarizations in single-element systems.

## Introduction

In the present era of high-end technology, electronic devices and communication facilities have become an dispensable part of nearly every aspect of daily life. However, the accompanying electromagnetic pollution problem has turned to be increasingly serious due to its damages to human beings and environment. Microwave absorption materials have drawn attention of researchers in industrial, commercial and military fields^1^, which could absorb or dissipate electromagnetic energy effectively^2^. The ideal microwave wave absorbers with merits of thin thickness, broad effective bandwidth, high absorptive capacity, low filler loading ratio and light weight are highly desirable at present^3,4^. To date, various microwave absorption materials have been successfully prepared, including dielectric loss materials, such as ZnO^5,6^, magnetic loss materials, for example Ni^7^, ferrite^8–10^ and composites materials such as CoFe_2_O_4_/graphene oxide^11^, Fe_3_O_4_@C^12^, Ni@SnO_2_^13^, Fe_3_O_4_/silica^14^, FeSn_2_/Sn/graphite^15^, CoNi@SiO_2_@TiO_2_^16^, which exhibit attracting microwave absorption performance.

Carbon allotropes and their derivatives have been extensively developed in the field of energy conversion due to their various forms, tunable physical and chemical properties and chemical stability^17–23^. Carbon-based microwave absorption materials, such as, porous carbon^24^, carbon fibers^25^, polyaniline^26^, carbon nanotubes (CNTs)^27,28^, graphene^5,29^, reduced graphene oxide (RGO)^30,31^, and carbon microspheres^3,32–35^, exhibit superior microwave absorption properties due to their dielectric attenuation capacity raised from the multiple dipolar configurations. Most often, the conventional synthetic strategies for carbon-based microwave absorption materials are constructing diverse micro/nanoscale architectures^27,29,32,33^, incorporating vacancies and heteroatoms^30,36^ and composing with heterogeneous components^24–26^. However, the relative research on synthetic strategy based on constructing isomeric heterogeneous interfaces is rarely reported.

In this paper, carbonized hydrochars composed of dispersed ultrafine nanocrystallines graphite and amorphous matrix have been successfully prepared on the basis of synthetic strategy of constructing isomeric heterogeneous interfaces. This unique isomeric construction with high-density interfaces between amorphous matrix and nanocrystalline graphite, accompanied with significant interfacial polarizations, leading to the improvement of microwave absorbing capabilities. Moreover, a very thin thickness of absorber can be achieved (minimum thickness for reflection loss less than −10 dB), comparing with other carbon-based materials, which manifests total weight of microwave absorption composites can be substantially lower for practical applications.

## Results and Discussion

**Figure 1:**
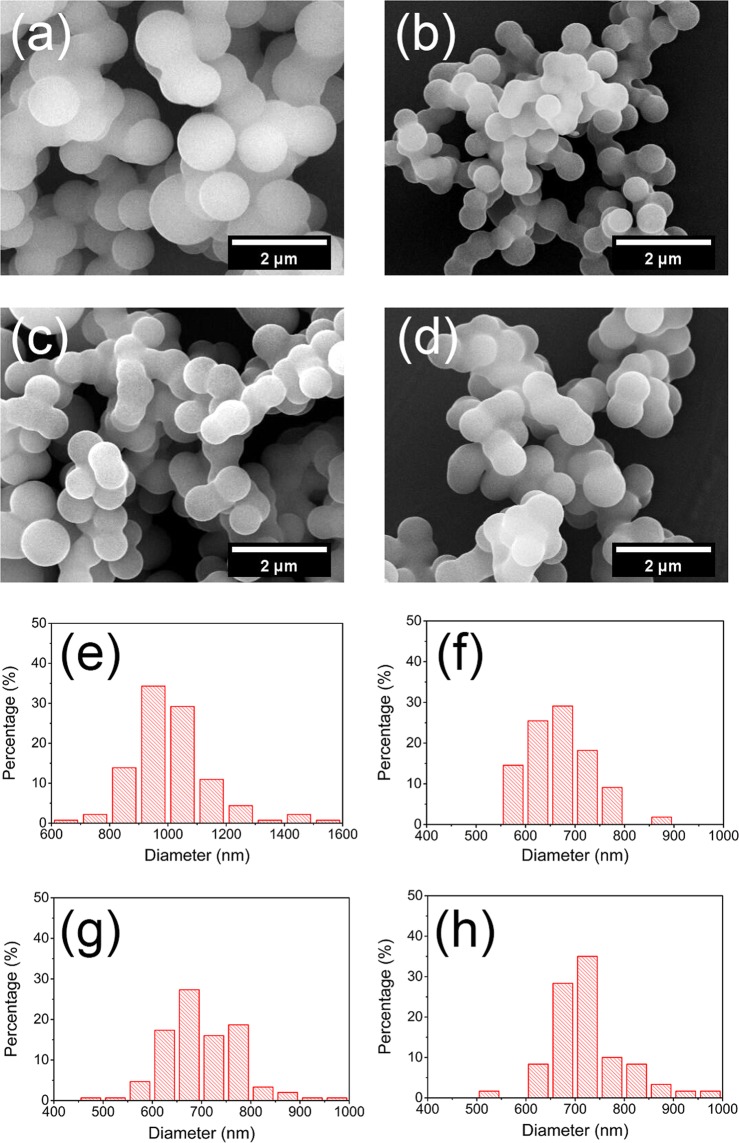
SEM images for (**a**) the original hydrochars, the carbonized hydrochars reacted at (**b**) 700 °C, (**c**) 800 °C and (**d**) 900 °C for 30 min, and corresponding diameter histograms of (**e**) the original hydrochars, the carbonized hydrochars reacted at (**f**) 700 °C, (**g**) 800 °C and (**h**) 900 °C for 30 min, respectively.

Initially, a series of experiments were conducted at 700 °C, 800 °C and 900 °C, while carbonized period was set to 30 min in this stage. The SEM images of hydrochars and carbonized products reacted at 700 °C, 800 °C, 900 °C for 30 min are shown in Fig. 1, the spherical morphologies of hydrochars and carbonized products could be clearly observed with some bonding particles, which are similar to each other. After carbonization, the mean diameters of products are around 700 nm, which are less than that of hydrochars, in accordance with size change trend of spherical activated carbons reported in previous literature^37,38^.

**Figure 2:**
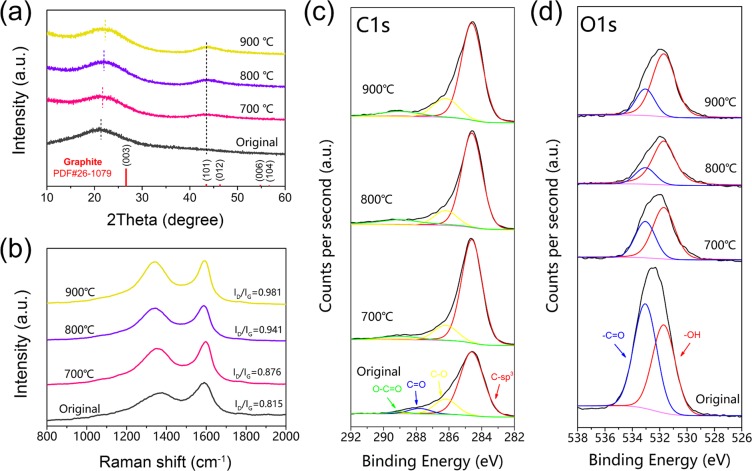
XRD spectra (**a**), Raman spectra (**b**), X-ray photoelectron spectra (**c**, C1s and **d**, O1s) of original hydrochars (Original) and carbonized hydrochars reacted at 700 °C, 800 °C, 900 °C for 30 min.

The XRD spectra of original hydrochars (designated as “Original” in Fig. 2), carbonized hydrochars reacted at 700 °C, 800 °C and 900 °C for 30 min are presented in Fig. 2a. It is obvious that the original hydrochars group shows a broad hump at 21.2°, which can be ascribed to amorphous carbons. However, there are two broad diffraction peaks at around 22° and 44° for all three types of carbonized hydrochars. It has been reported that small domains of stacked crystalline graphite can be formed after high temperature carbonization, whose diffraction peaks locate at 24.3° and 44.8°^39^. For all three types of carbonized hydrochars mentioned above, the peaks at around 22° results from existence of nanocrystallines graphite and amorphous carbons after carbonization, while the peak at around 44° corresponds to the graphite (101) plane (JCPDS 26-1079). Besides, the peak at around 22° moves towards large angle with the increase of carbonized temperature (indicated by dashed lines in Fig. 2a), indicates an increase in amount of nanocrystalline graphite.

Figure 2b shows the Raman spectra of original hydrochars, carbonized hydrochars reacted at 700 °C, 800 °C and 900 °C for 30 min. All four spectra show two different peaks, the one located at around 1350 cm^−1^ (D band), while the other centered at 1590 cm^−1^ (G band). Herein, the I_D_/I_G_ value increases with carbonized temperature from 0.815 (Original) to 0.981 (900 °C). As previously reported literature^40–42^, near K zone boundary, D band resulted from A_1g_ symmetry breathing mode involving phonons is active with the existence of nanocrystalline graphite or disorder; G band resulted from E_2g_ symmetry mode originates from in-plane bond-stretching motion of carbon sp^2^ atom pairs. Besides, Ferrari *et al*. brought forward a transition model from graphite to amorphous carbon (ta-C) based on changes of I_D_/I_G_, and the enlarged I_D_/I_G_ value is derived from evolution of amorphous carbon to nanocrystalline graphite^42^. In other word, enlarged value of I_D_/I_G_ at carbonized temperature indicates the amount of nanocrystalline graphite increases for carbonized hydrochars, which is exactly consistent with XRD results above.

Further details of the chemical structure of original hydrochars, carbonized hydrochars reacted at 700 °C, 800 °C and 900 °C were conducted, as shown in Fig. 2c,d. The C 1 s spectra are fitted with C-C, C-O, C=O and O-C=O functional groups, located at 284.6, 286.2, 287.8 and 289.0 eV, respectively^3,5,43^. For all three types of carbonized hydrochars, no signal of C=O functional group has been detected. Besides, their corresponding reducing signal areas of oxygen-containing functional groups (C-O, C=O, O-C=O), as well as enlarging signal area of C-C functional group indicate relative contents of oxygen-containing functional groups decrease, compared with signal areas of original hydrochars. And the O 1 s spectra could be deconvoluted into two individual component peaks at 533.1 and 531.7 eV, corresponding to the -C-O, -O-H bonds^3,44^. From Fig. 2d, we could also observe the obvious signal area changes of both -C-O and -O-H functional groups between original hydrochars and three types of carbonized hydrochars. More specifically, the relative content of -C-O functional groups drastically decreases after high temperature carbonization. These results above are closely interrelated with transition from amorphous carbon to nanocrystalline graphite.

**Figure 3:**
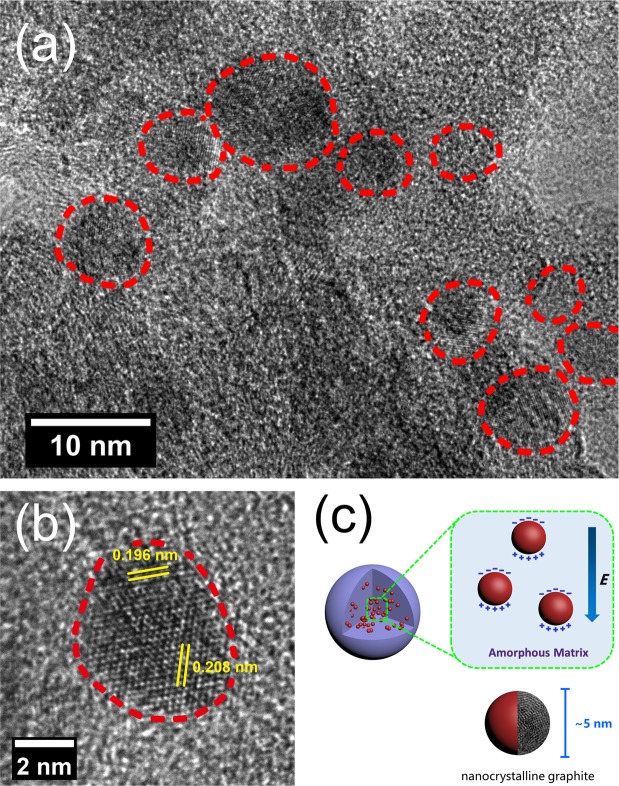
Typical HRTEM images of carbonized hydrochars reacted at 800 °C for 20 min (**a**,**b**) and schematic illustration of carbonized hydrochars (**c**).

It has been reported that small domains of stacked crystalline graphite are extremely difficult to be detected by TEM technique^39^. Herein, carbonized hydrochars reacted at at 800 °C for 20 min were selected for TEM characterization by means of ultrathin sections with the thickness of 40 nm. During process of TEM characterization, we found that nanocrystallines graphite were uniformly dispersed in carbonized hydrochars with sizes slightly varying from each other and that amorphous carbons without crystallization in large amount could be observed. The typical HRTEM images of carbonized hydrochars reacted at 800 °C for 20 min are shown in Fig. 3a,b. Figure 3a provides a clear view of nanocrystalline graphite particles with size of about 5 nm, which marked by red dotted line. And Fig. 3b, obtained from higher magnification, reveals that corrugated parallel fringes with basal spacing values of 0.196 nm and 0.208 nm depict the lattice-resolved (012) and (101) crystalline planes of graphite structure. Moreover, some small black regions with incomplete crystallization and lattice distortions resulted from atom dislocations could be clearly observed in Fig. 3a,b, respectively, indicating many defects existed in the domains of nanocrystalline graphite, which could be polarized centers. And the TEM results above are coincident with the XRD patterns of carbonized samples. More specifically, the broadening of (101) graphite diffraction peak has a strong relationship with defects in the domains of nanocrystalline graphite during the transition from amorphous carbons to crystalline graphites.

**Figure 4:**
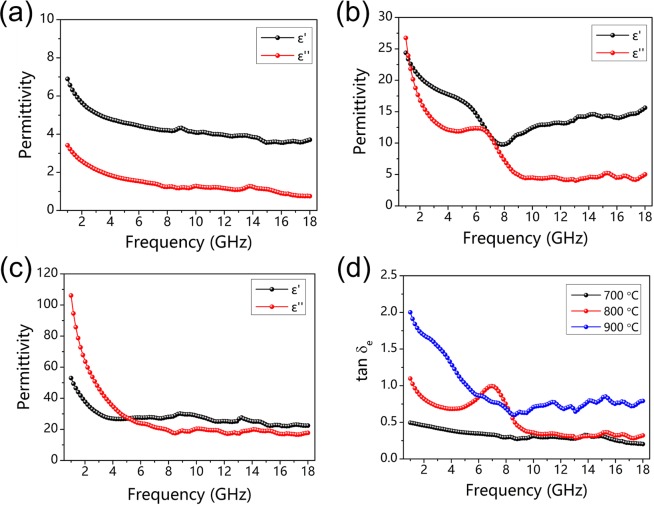
The complex permittivity for carbonized hydrochars reacted at (**a**) 700 °C, (**b**) 800 °C, (**c**) 900 °C for 30 min, and (**d**) their corresponding dielectric dissipation factors (tan δ_e_ = ε″/ε′).

The microwave absorption performance of carbonized hydrochars reacted at 700 °C, 800 °C and 900 °C for 30 min were investigated by measuring relative complex permittivity (*ε*_*r*_ = *ε*′ − j*ε*″) as well as relative complex permeability (*μ*_*r*_ = *μ*′ − j*μ*″). And corresponding real and imaginary permittivities are presented in Fig. 4a–c, *ε*′ values of three samples are in the range of 6.9–3.5, 24.4–9.8 and 53.0–22.4, respectively, indicating a decreased tendancy with increased frequency. Similarly, *ε*″ values of three samples decrease along with the increasing frequency, and they are in the range of 3.4–0.8, 26.7–4.0 and 106.1–16.6, respectively. And their corresponding dielectric dissipation factors (tan*δ*_*e*_ = *ε*″/*ε*′) are presented in Fig. 4d. In contrast to the features of complex permittivity, complex permeability for three samples show little change ranged from 1 to 18 GHz. And the complex permeability for all three types of carbonized hydrochars are close to unity (*μ*′ and *μ*″ at around 1 and 0), as shown in Fig. S1 (Supporting Information).

From the transmission line theory, the reflection loss (RL) values of microwave can be acquired by measuring the complex permittivity and complex permeability^45^,

1 $${Z}_{in}={Z}_{0}\sqrt{\frac{{\mu }_{r}}{{\varepsilon }_{r}}}tanh[j(\frac{2\pi fd}{c})\sqrt{{\mu }_{r}{\varepsilon }_{r}}]$$

2 $${\rm{RL}}\,(dB)=20log|\frac{{Z}_{in}-{Z}_{0}}{{Z}_{in}+{Z}_{0}}|$$

where *Z*_*in*_ is the normalized input impedance, *Z*_0_ represents the impedance of free space, *ε*_*r*_ and *μ*_*r*_ refer to the complex permittivity and complex permeability, respectively, *f* is the frequency of microwave, *d* is the absorber thickness, and *c* is the velocity of light.

**Figure 5:**
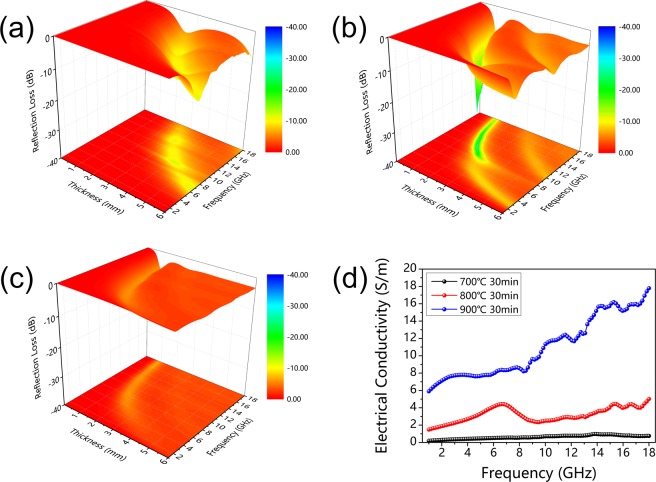
The 3D graphs of RL values for carbonized hydrochars reacted at (**a**) 700 °C, (**b**) 800 °C, and (**c**) 900 °C for 30 min, (**d**) plots of electrical conductivity versus frequency of carbonized hydrochars in the alternating electromagnetic field.

The 3D graphs of RL values for carbonized hydrochars with different thickness with paraffin loading ratio of 50 wt.% are displayed in Fig. 5a–c, and RL curves with different thicknesses are shown in Fig. S2 (Supporting Information). It can be found that the minimums of RL value for all three types of carbonized hydrochars shift toward lower frequency with increased thickness, which can be explained by quarter-wavelength cancellation model^7,46,47^. For the hydrochars carbonized at 700 °C for 30 min, the minimum RL value achieves −12.08 dB with the thickness of 4.12 mm (9.84 GHz). The RL values of carbonized hydrochars reacted at 900 °C with arbitrary thickness don’t exceed −10 dB with frequency range from 1 to 18 GHz (minimum: −6.17 dB at 16.30 GHz). It is noteworthy that the carbonized hydrochars reacted at 800 °C exhibits excellent absorption performance towards microwaves, especially at 10.35 GHz, the minimum RL value can achieve −28.20 dB with 2.02 mm thickness, and corresponding effective bandwidth (RL value <−10 dB, 90% microwave attenuation) reaches 2.1 GHz.

The ability of dielectric loss originated from polarization loss and conductivity loss has been widely accepted, and the loss of polarization includes interfacial polarization, dipole orientation polarization, ionic polarization and electronic polarization^48^. Usually electronic polarization and ionic polarization are found at high frequency range (10^3^–10^6^ GHz), so their effects could be easily eliminated^48^. Herein, isomeric heterogeneous interfacial polarizations as well as dipole polarizations resulted from incomplete crystallization and lattice distortions contribute enormously to microwave absorption. The specific isomeric structure guarantees induced charges accumulation on the interfaces between amorphous matrix and nanocrystalline graphite. Under alternating electromagnetic field, it is favorable for induced charges to migrate to response external electromagnetic field, which results in electromagnetic energy loss.

According to the free electron theory^49–52^, the electrical conductivity of absorbers can be simply estimated based on the equation as below:

3 $$\varepsilon ^{\prime\prime} =\frac{\sigma }{\omega {\varepsilon }_{0}}=\frac{\sigma }{2\pi f{\varepsilon }_{0}}\to \sigma =2\pi f{\varepsilon }_{0}\varepsilon ^{\prime\prime} $$

where *f* is frequency, *ε*_0_ represents the permittivity of free space (*ε*_0_ = 8.854 × 10^−12^ *F*·*m*^−1^), and *ε*″ is the imaginary permittivity. Figure 5d exhibits the calculated electrical conductivity of above three absorbers in alternating electromagnetic field. Apparently, the carbonized hydrochars reacted at 700 °C for 30 min presents the lowest electrical conductivity with no more than 1 S m^−1^, and the carbonized hydrochars reacted at 900 °C has relatively high conductivity (6–18 S m^−1^). Both two types of carbonized hydrochars above show relatively poor microwave absorption performance. This is because lower carbonized temperature or shorter carbonized period would reduce the degree of removal of oxygen-containing functional groups and decrease the amount of isomeric heterogeneous interfaces between amorphous matrix and nanocrystallines graphite, which have an adverse effect on isomeric heterogeneous interfacial polarizations and dipole polarizations, leading to poor microwave absorption property. And for higher carbonized temperature case, only high complex permittivity (*ε*′ and *ε*″) could not obtain outstanding microwave absorbing performance when considering the matched characteristic impedance. The matched characteristic impedance of absorbers should be equal to 377 Ω sq^−1^ for the achievement of zero-reflection at the materials front surface^33,49^. In general, the distinction of complex permeability and permittivity does harm to the matched characteristic impedance, which leads to a strong reflection of incident microwave^33,43^. The above results illustrate that electrical conductivity within an appropriate range is extremely crucial for absorbers in this work, high electrical conductivity would lead to impedance mismatch, and the weak isomeric heterogeneous interfacial polarizations and dipole polarizations are inevitable when corresponding electrical conductivity is relatively low.

**Figure 6:**
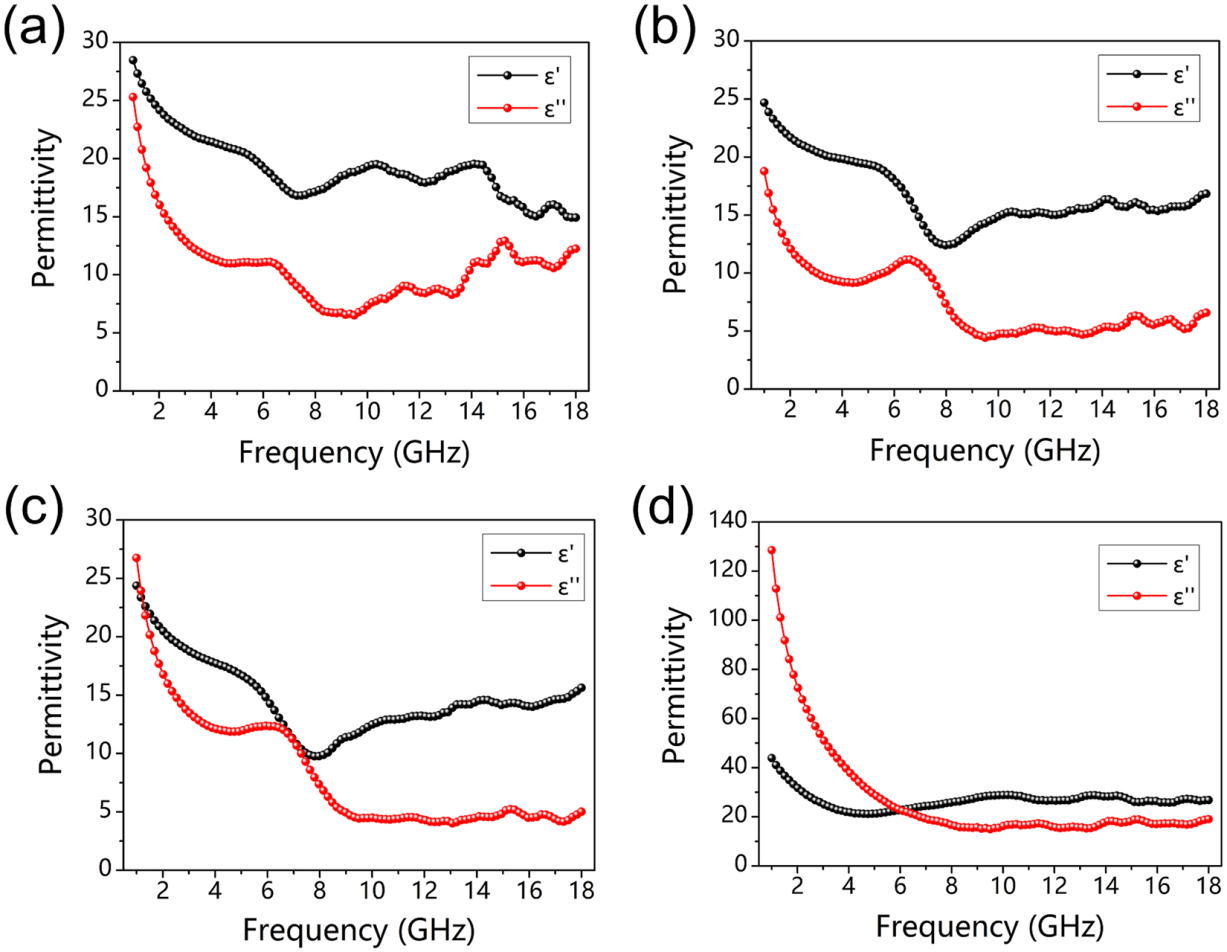
The complex permittivity for carbonized hydrochars reacted at 800 °C for (**a**) 15 min, (**b**) 20 min, (**c**) 30 min and (**d**) 40 min.

In order to further optimize the experimental parameters, herein, four samples of hydrochars were selected in this experimental group under different carbonized periods (15, 20, 30 and 40 min), while carbonized temperature maintained at 800 °C. As mentioned above, all these four samples after carbonization show *ε*′ and *ε*″ at around 1 and 0 (not shown). Obviously, as shown in Fig. 6, complex permittivity curves for carbonized hydrochars reacted for 15, 20, 30 and 40 min vary from each other, and there are some fluctuations for *ε*′ and *ε*″ in high frequency range. The *ε*′ values of corresponding sample are 28.5–14.8, 24.8–12.3, 24.4–9.8 and 43.9–21.2, while their *ε*″ values are in the range of 25.4–6.5, 18.9–4.3, 26.7–4.0 and 128.6–15.4, respectively.

**Figure 7:**
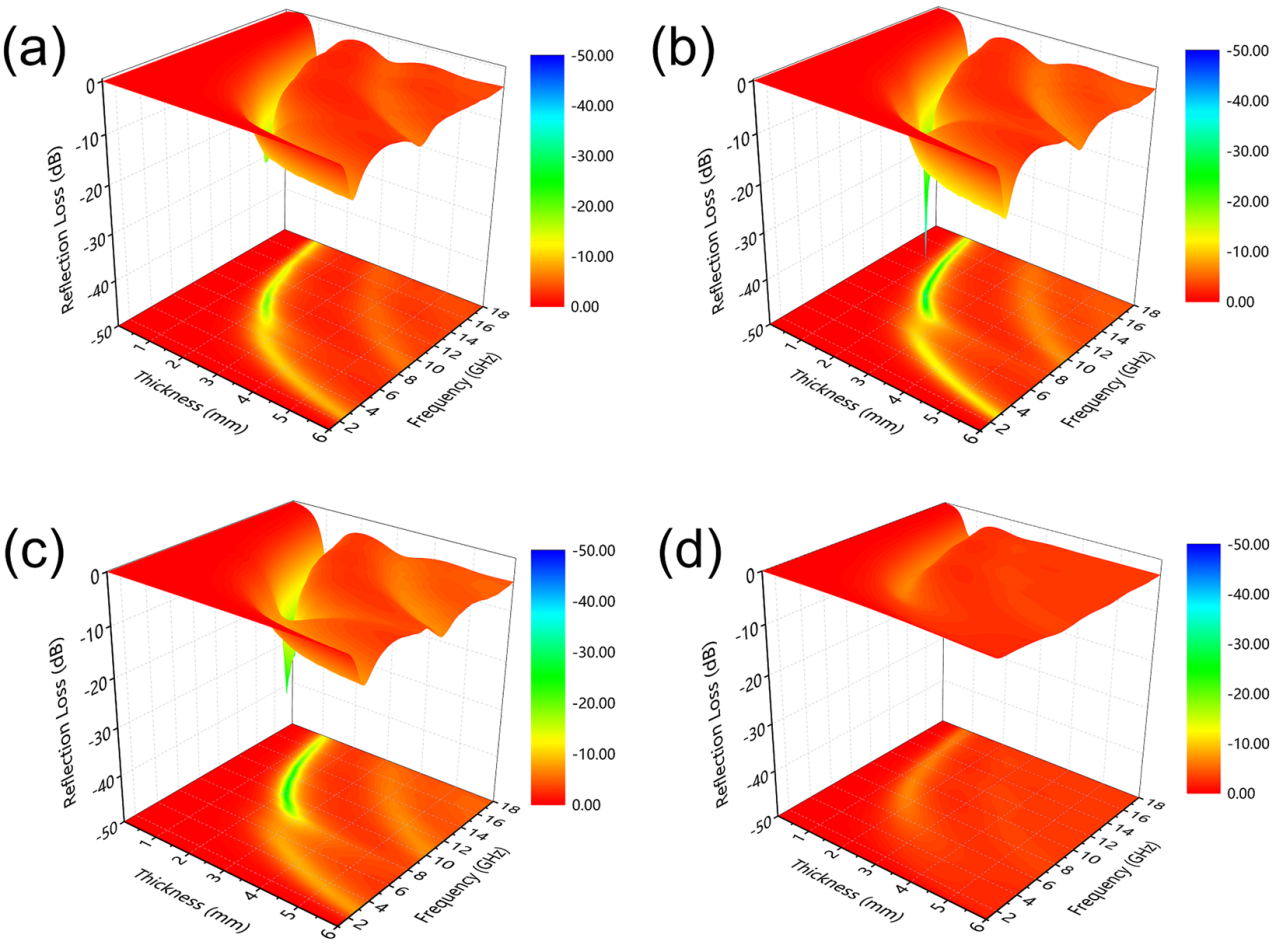
The 3D graphs of RL values for carbonized hydrochars reacted at 800 °C for (**a**) 15 min, (**b**) 20 min, (**c**) 30 min and (**d**) 40 min.

The 3D graphs of RL values and RL curves with different thicknesses for carbonized hydrochars reacted at 800 °C for 15, 20, 30 and 40 min are clearly shown in Figs 7 and S3 (Supporting Information), respectively. As observed, the minimum RL value of the hydrochars carbonized for 40 min could not reach −10 dB (90% microwave attenuation). Most noteworthy, carbonized hydrochars reacted for 20 min exhibits obviously enhanced microwave absorbing capability, especially at absorber thickness of 1.93 mm, the RL_min_ value can achieve −40.36 dB at 10.18 GHz, and their effective bandwidth is 2 GHz (9.3–11.3 GHz), as shown in Fig. S3b. As for carbonized hydrochars reacted for 15 min, their minimum RL value is −19.58 dB (9.50 GHz, thickness 1.91 mm), and their corresponding effective bandwidth is 1.7 GHz (8.6–10.3 GHz). Therefore, the hydrochars carbonized for 20 min exhibits outstanding microwave absorption performance among absorbers in this work, and its absorption properties could be modified by varying the thickness of absorbers in practical applications.

**Figure 8:**
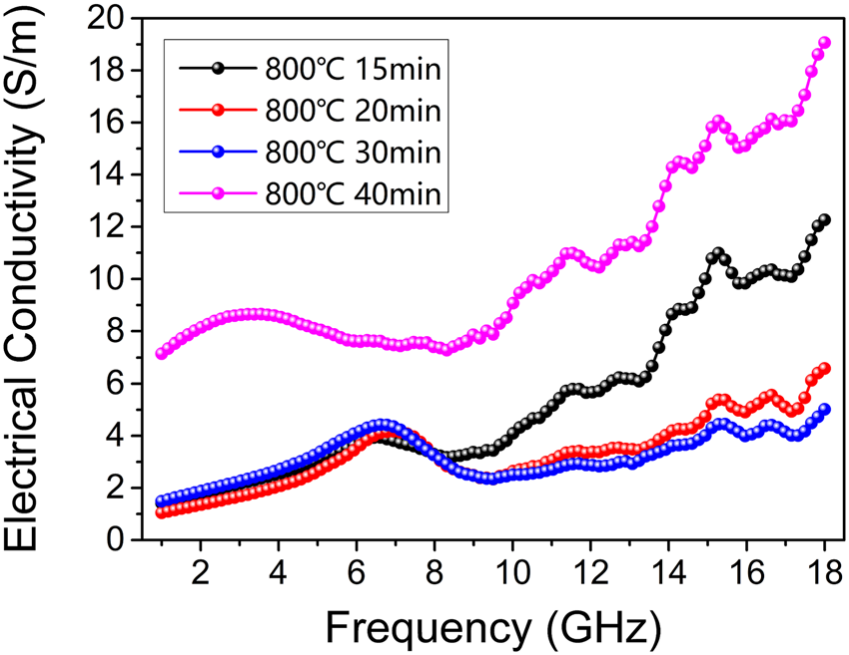
The plots of electrical conductivity versus frequency of carbonized hydrochars reacted at 800 °C for different periods in the alternating electromagnetic field.

Figure 8 exhibits the calculated electrical conductivity of carbonized hydrochars reacted at 800 °C for different periods in alternating electromagnetic field. Some absorbers in this work (carbonized hydrochars reacted for 20 min and 30 min) show better absorption performances, and their electrical conductivity curves show similar increasing trend in the measured frequency range with values ranging from 1 to 7 S m^−1^. As mentioned above, electrical conductivity within an appropriate range is extremely crucial for absorbers, which is closely associated with the amount of isomeric heterogeneous interfaces between nanocrystalline graphite and amorphous matrix.

An interesting result has been discovered that under the condition of low RL values (below −10 dB), the minimum thicknesses for absorber carbonized at 800 °C for 20 min are thinner than those of many absorbers in previous literature at X and Ku bands (8–18 GHz). The minimum thicknesses for different absorbers and that obtained in this work (RL values below −10 dB) are listed in Table 1, which are investigated by measuring the contours of RL values from these absorbers. The thinner thickness is conductive to reduce the structure weight of absorbers, which is favorable to practical application. Besides, the hydrochars as precursors can be synthesized from biomass (such as glucose, sucrose, starch, cellulose etc.)^38,53,54^, which are available in abundance with low cost. Additionally, only two simple and controllable steps including hydrothermal carbonization of biomass and high temperature carbonization are needed to prepare absorbers in this work. Undoubtedly, due to all the merits mentioned above, carbonized hydrochars are promising and appealing materials in the area of microwave absorption, especially for composing other dielectric or magnetic materials.

**Table 1:** The minimum thickness of various absorbers in the frequency range of 8–18 GHz (RL values <-10 dB).

Filler	Matrix	Filer loading [%]	Frequency [GHz]						Ref.
			8	10	12	14	16	18	
Carbonized hydrochars	Paraffin	50	2.35^a^	1.76	1.44	1.22	1.08	0.98	Herein
Yolk-Shell-Fe_3_O_4_@C	Paraffin	70	2.94	2.45	2.10	1.82	1.62	1.50	^12^(2017)
Biomass-derived CS	Paraffin	25	—	—	2.06	1.64	1.60	1.61	^36^(2015)
PCHMs	Phenolic resin	20	2.71	2.15	1.81	1.88	1.72	1.56	^3^(2017)
Ni@SiC	Paraffin	60	2.91	2.53	2.29	1.93	1.54	1.41	^7^(2017)

^a^Thickness measured in millimeters [mm].

## Conclusions

In conclusion, we successfully prepared carbonized hydrochars composed of dispersed ultrafine nanocrystallines graphite and amorphous matrix on the basis of synthetic strategy of constructing isomeric heterogeneous interfaces. The isomeric heterogeneous interfacial polarizations as well as dipole polarizations resulted from incomplete crystallization and lattice distortions contribute enormously to microwave absorption. This study demonstrates a micro-interface scale insight to engineer microwave absorption materials and can be promised to have great potential for designing and manufacturing novel microwave-absorbing devices.

## Methods

### Synthesis of carbonized hydrochars

The synthetic procedure of hydrochars was carried out by the hydrothermal method^53^. Experimentally, 0.5 M sucrose solution (60 ml) was transferred to a Teflon-lined stainless steel autoclave with 80 ml and then heated for 12 h at 180 °C. The brown precipitates were collected by centrifugation and abstersion, followed by placed in vacuum oven at 80 °C overnight.

Subsequently, 5 g hydrochars were transferred to a lidded corundum crucible (30 ml), and then heated in a tube furnace with argon gas (Ar) protection. In the first set, tube furnace was respectively heated up to 700, 800, 900 °C for 30 min, the heating speed maintaining 5 °C min^−1^. And tube furnace was heated to 800 °C for 15, 20, 30 and 40 min in the second set of experiments. The argon gas protection were applied in both heating process and natural cooling process.

### Materials characterization

The morphologies of samples were observed by field emission scanning electron microscope (Zeiss Ultra 55), and the sizes of samples and statistic analysis were obtained by image processing software. X-ray diffraction (XRD) characterization were conducted on a diffractometer (Rigaku D/max-A) with Cu Kα radiation (λ = 1.5406 Å). Photoelectron spectrometer (Thermo ESCALAB 250) equiped with monochromatic Al Kα radiation at 1486.6 eV was used to collect the XPS spectra, and the C 1 s binding energy was calibrated at 284.6 eV for the high-resolution spectra. At the excitation wavelength of 532 nm, the Raman spectra were collected on a microscope (HORIBA XploRA PLUS). The ultrathin section was selected for carbonized hydrochars on a ultramicrotome (Leica EM UC 6), in order to conduct TEM characterizations. The microstructure of samples were characterized by a transmission electron microscope (JEOL JEM-ARM 200 F).

For preparation of microwave absorption test, the paraffins with 50 wt.% carbonized hydrochars powders were mixed in the samples. Then the mixture was pressed into a standard toroidal shape (Φ_in_ = 3.04 mm, Φ_out_ = 7.00 mm). The vector network analyzer (Agilent E5071C) in the frequency range of 1 to 18 GHz was used to acquire the complex permittivity as well as the complex permeability of samples with coaxial line.

## Supplementary information

Supplementary Materials
