# Supplementary material for: Amorphous/Nanocrystalline Carbonized Hydrochars with Isomeric Heterogeneous Interfacial Polarizations for High-performance Microwave Absorption
Source: Sci Rep. 2019 Aug 27;9:12429. doi: 10.1038/s41598-019-48926-3 (PMC6712053; doi:10.1038/s41598-019-48926-3)
Supplement: Supplementary file 1 — Supplementary Materials [file 41598_2019_48926_MOESM1_ESM.doc]

***Supporting Information***

Amorphous/Nanocrystalline Carbonized Hydrochars with Isomeric Heterogeneous Interfacial Polarizations for High-performance Microwave Absorption

Yujie Qi, Dongchao Wei, Gui-Mei Shi*, Mu Zhang, Yang Qi*


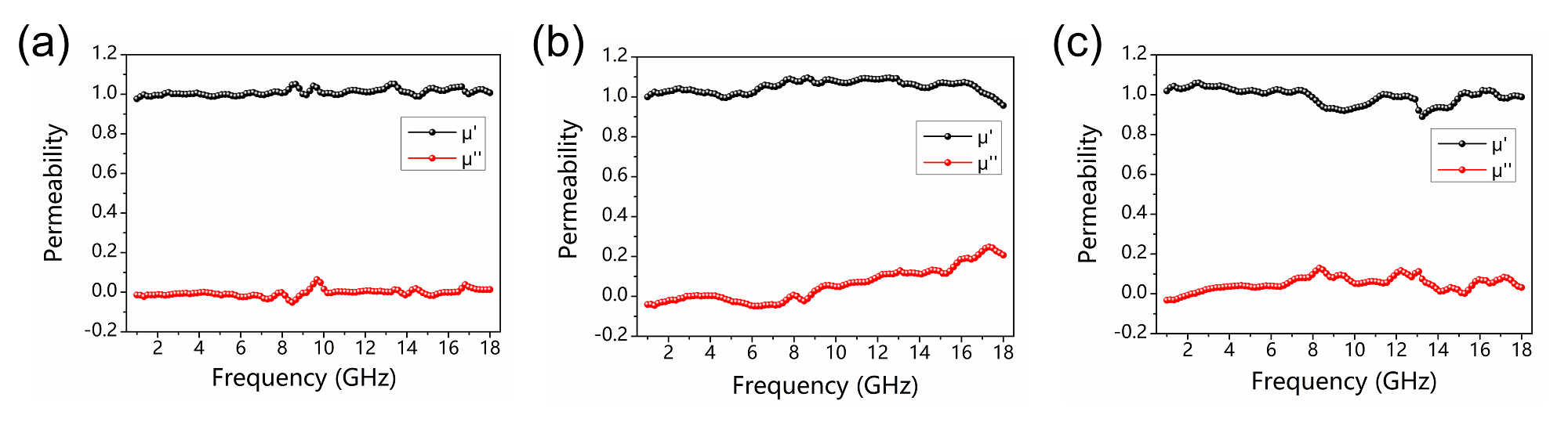


**Fig. S1.** Real part and imaginary part of the complex permeability of carbonized hydrochars reacted at (a) 700 °C, (b) 800 °C, and (c) 900 °C for 30 min.


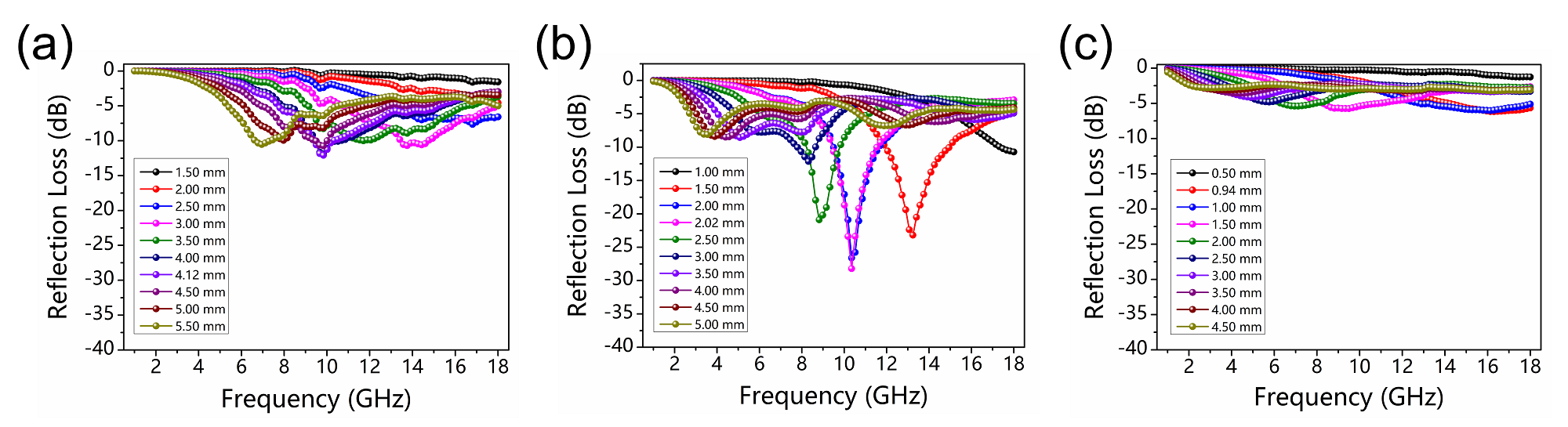


**Fig. S2.** Reflection loss curves of carbonized hydrochars reacted at (a) 700 °C, (b) 800 °C, and (c) 900 °C for 30 min with different thicknesses.


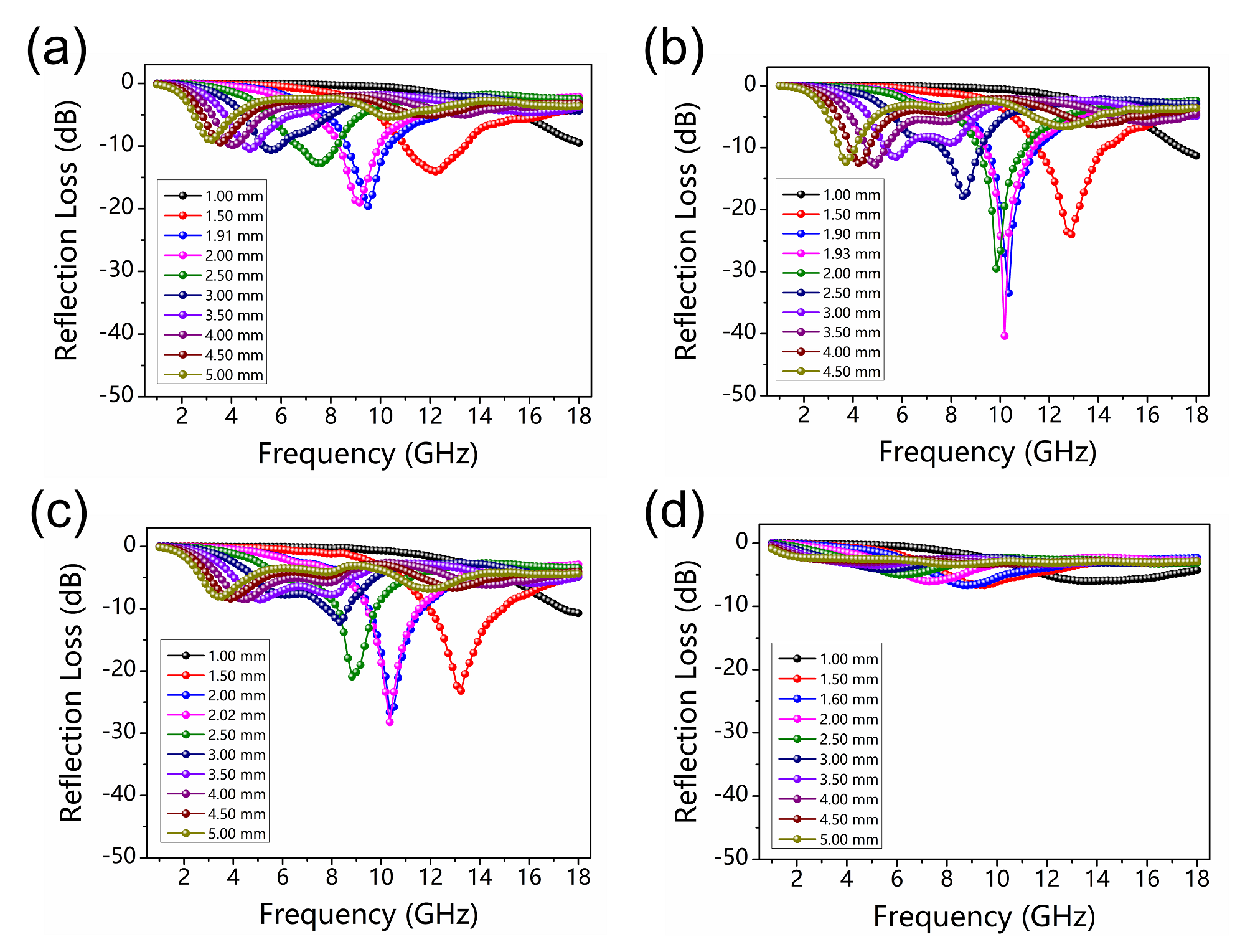


**Fig. S3**. Reflection loss curves of carbonized hydrochars reacted at 800 °C for (a) 15 min, (b) 20 min, (c) 30 min and (d) 40 min with different thicknesses.

**Table S1**. Reflection loss characteristics of absorbers in this work

| Entry | Carbonized temperature [°C] | Carbonized period [min] | Thickness [mm] | RL_max_ [dB] | Frequency range [GHz]  (RL below -10 dB) | Effective bandwidth [GHz]  (RL below -10 dB) |
| --- | --- | --- | --- | --- | --- | --- |
| 1 | 700 | 30 | 4.12 | -12.08 | 9.4-10.3 | 0.9 |
| 2 | 800 | 30 | 2.02 | -28.20 | 9.4-11.5 | 2.1 |
| 3 | 900 | 30 | 0.94 | -6.17 | - | - |
| 4 | 800 | 15 | 1.91 | -19.58 | 8.6-10.3 | 1.7 |
| 5 | 800 | 20 | 1.93 | -40.36 | 9.3-11.3 | 2 |
| 6 | 800 | 40 | 1.60 | -6.65 | - | - |
